# Supplementary material for: Insight into postural control in unilateral sensorineural hearing loss and vestibular hypofunction
Source: PLoS One. 2022 Oct 17;17(10):e0276251. doi: 10.1371/journal.pone.0276251 (PMC9576045; doi:10.1371/journal.pone.0276251)
Supplement: S1 Table — (DOCX) [file pone.0276251.s002.docx]

**S1 Table**: Analysis confirming removal of sound as a predictor

| **Variable** | **AIC No Sound (3-way interaction)** | **AIC With Sound (4-way interaction)** |
| --- | --- | --- |
| DP ML COP | -236.2616 | -166.4970 |
| DP AP COP | -512.4857 | -437.0875 |
| DP ML Head | -196.2672 | -131.0197 |
| DP AP Head | -495.4569 | -422.6889 |
| DP Pitch Head | -541.4461 | -472.5920 |
| DP Yaw Head | -526.9042 | -458.3456 |
| DP Roll Head | -655.7581 | -582.3711 |
