# Supplementary material for: Insight into postural control in unilateral sensorineural hearing loss and vestibular hypofunction
Source: PLoS One. 2022 Oct 17;17(10):e0276251. doi: 10.1371/journal.pone.0276251 (PMC9576045; doi:10.1371/journal.pone.0276251)
Supplement: S2 Table — (DOCX) [file pone.0276251.s003.docx]

**S2 Table**

**Model point estimates and their 95% Confidence Intervals [CI] per outcome, group and condition.**

|  | **Control** | **Vestibular** | **USNHL** |
| --- | --- | --- | --- |
| **COP Medio-lateral (cm) model estimates and 95% CI** | | | |
| **Floor Static** | 27.7 [24.5, 31.4] | 37.4 [31.8, 44.0] | 30.8 [25.5, 37.2] |
| **Floor Dynamic** | 29.0 [25.6, 32.8] | 40.1 [34.0, 47.1] | 30.9 [25.6, 37.3] |
| **Foam Static** | 66.6 [58.8, 75.2] | 98.2 [83.3, 115.8] | 64.8 [53.7, 78.2] |
| **Foam Dynamic** | 76.5 [67.6, 86.5] | 116.5 [98.8, 137.4] | 68.6 [56.9, 82.8] |
| **COP Anterior-posterior (cm) model estimates and 95% CI** | | | |
| **Floor Static** | 52.2 [46.3, 58.9] | 65.7 [56.0, 77.1] | 49.7 [41.3, 59.8] |
| **Floor Dynamic** | 59.7 [52.9, 67.4] | 77.4 [65.9, 90.8] | 53.4 [44.4, 64.2] |
| **Foam Static** | 97.6 [86.5, 110.2] | 157.7 [134.2, 185.3] | 88.4 [73.5, 106.4] |
| **Foam Dynamic** | 141.4 [125.2, 159.5] | 238.1 [202.5, 279.9] | 115.2 [95.7, 138.5] |
| **Head Medio-lateral (cm) model estimates and 95% CI** | | | |
| **Floor Static** | 17.2 [15.4, 19.3] | 25.7 [22.1, 29.7] | 20.6 [17.3, 24.4] |
| **Floor Dynamic** | 17.5 [15.7, 19.6] | 27.0 [23.3, 31.3] | 20.0 [16.9, 23.7] |
| **Foam Static** | 39.6 [35.4, 44.3] | 59.6 [51.2, 69.2] | 44.4 [37.4, 52.6] |
| **Foam Dynamic** | 43.7 [39.0, 48.8] | 67.5 [58.0, 78.5] | 44.6 [37.6, 52.9] |
| **Head Anterior-posterior (cm) model estimates and 95% CI** | | | |
| **Floor Static** | 28.9 [26.2,31.9] | 39.4 [34.6, 44.9] | 31.9 [27.5, 37.1] |
| **Floor Dynamic** | 34.5 [31.2, 38.1] | 49.5 [43.4, 56.4] | 35.9 [30.8, 41.7] |
| **Foam Static** | 49.3 [44.7, 54.4] | 75.9 [66.5, 86.7] | 51.5 [44.7, 60.4] |
| **Foam Dynamic** | 70.4 [63.8, 77.7] | 113.8 [99.6, 130.0] | 68.2 [58.7, 79.4] |
| **Head Pitch (rad) model estimates and 95% CI** | | | |
| **Floor Static** | 0.74 [0.65, 0.85] | 0.86 [0.72, 1.02] | 0.71 [0.58, 0.88] |
| **Floor Dynamic** | 0.78 [0.68, 0.89] | 0.90 [0.75, 1.07] | 0.72 [0.59, 0.88] |
| **Foam Static** | 1.00 [0.89, 1.15] | 1.59 [1.33, 1.89] | 0.96 [0.78, 1.17] |
| **Foam Dynamic** | 1.14 [1.00, 1.31] | 2.05 [1.72, 2.45] | 1.06 [0.87, 1.30] |
| **Head Yaw (rad) model estimates and 95% CI** | | | |
| **Floor Static** | 0.71 [0.64, 0.79] | 0.81 [0.71, 0.93] | 0.64 [0.55, 0.75] |
| **Floor Dynamic** | 0.72 [0.65, 0.80] | 0.80 [0.70, 0.91] | 0.60 [0.52, 0.71] |
| **Foam Static** | 0.99 [0.89, 1.10] | 1.29 [1.12, 1.45] | 0.93 [0.79, 1.09] |
| **Foam Dynamic** | 1.07 [0.97, 1.19] | 1.47 [1.28, 1.68] | 0.97 [0.83, 1.14] |
| **Head Roll (rad) model estimates and 95% CI** | | | |
| **Floor Static** | 0.54 [0.49, 0.60] | 0.60 [0.53, 0.69] | 0.54 [0.47, 0.63] |
| **Floor Dynamic** | 0.54 [0.49, 0.59] | 0.59 [0.52, 0.67] | 0.53 [0.46, 0.61] |
| **Foam Static** | 0.72 [0.66, 0.80] | 0.96 [0.84, 1.09] | 0.75 [0.65, 0.86] |
| **Foam Dynamic** | 0.76 [0.69, 0.84] | 1.10 [0.96, 1.25] | 0.75 [0.64, 0.86] |
