# Supplementary material for: Insight into postural control in unilateral sensorineural hearing loss and vestibular hypofunction
Source: PLoS One. 2022 Oct 17;17(10):e0276251. doi: 10.1371/journal.pone.0276251 (PMC9576045; doi:10.1371/journal.pone.0276251)
Supplement: Attachment — Submitted filename: PONE RESPONSE.docx [file pone.0276251.s004.docx]

**Summary of Revisions**

**PONE-D-22-14616**

Insight into postural control in unilateral sensorineural hearing loss and vestibular hypofunction

PLOS ONE

Dear Dr. Monsanto and fellow referees,

We thank you for a thorough, detailed, constructive and very helpful review. We have made all of the corrections and revised the manuscript according to your suggestions. Below is a point-by-point list detailing how each of your comments was addressed.

Sincerely,

The Authors

***************************************************************************

Journal Requirements:

When submitting your revision, we need you to address these additional requirements.

1. Please ensure that your manuscript meets PLOS ONE's style requirements, including those for file naming. The PLOS ONE style templates can be found at 
[https://journals.plos.org/plosone/s/file?id=wjVg/PLOSOne_formatting_sample_main_body.pdf](https://urldefense.com/v3/__https:/journals.plos.org/plosone/s/file?id=wjVg*PLOSOne_formatting_sample_main_body.pdf__;Lw!!BhJSzQqDqA!XycIuz7H1Nut8UzwslsRAufUGRLNua0ZSRCnaf6zTUshw5rE0s4ct8duOg8he9Ma-xg4HuYbdI8P$) and 
[https://journals.plos.org/plosone/s/file?id=ba62/PLOSOne_formatting_sample_title_authors_affiliations.pdf](https://urldefense.com/v3/__https:/journals.plos.org/plosone/s/file?id=ba62*PLOSOne_formatting_sample_title_authors_affiliations.pdf__;Lw!!BhJSzQqDqA!XycIuz7H1Nut8UzwslsRAufUGRLNua0ZSRCnaf6zTUshw5rE0s4ct8duOg8he9Ma-xg4HpX_Dyxt$).

**Done.**

2. Please ensure that you include a title page within your main document. We do appreciate that you have a title page document uploaded as a separate file, however, as per our author guidelines ([http://journals.plos.org/plosone/s/submission-guidelines#loc-title-page](https://urldefense.com/v3/__http:/journals.plos.org/plosone/s/submission-guidelines*loc-title-page__;Iw!!BhJSzQqDqA!XycIuz7H1Nut8UzwslsRAufUGRLNua0ZSRCnaf6zTUshw5rE0s4ct8duOg8he9Ma-xg4Hq8SzUC5$)) we do require this to be part of the manuscript file itself and not uploaded separately.
Could you therefore please include the title page into the beginning of your manuscript file itself, listing all authors and affiliations.

**Done.**

3. Please provide additional details regarding participant consent. In the ethics statement in the Methods and online submission information, please ensure that you have specified what type you obtained (for instance, written or verbal, and if verbal, how it was documented and witnessed). If your study included minors, state whether you obtained consent from parents or guardians. If the need for consent was waived by the ethics committee, please include this information.

**Done. Participants provided a written informed consent. No minors were included in the study.**

4. We note that the grant information you provided in the ‘Funding Information’ and ‘Financial Disclosure’ sections do not match.

When you resubmit, please ensure that you provide the correct grant numbers for the awards you received for your study in the ‘Funding Information’ section.

**Grant numbers were added as applicable.**

5. We note that you have stated that you will provide repository information for your data at acceptance. Should your manuscript be accepted for publication, we will hold it until you provide the relevant accession numbers or DOIs necessary to access your data. If you wish to make changes to your Data Availability statement, please describe these changes in your cover letter and we will update your Data Availability statement to reflect the information you provide.

**The data are available here:**

[**https://data.mendeley.com/datasets/kcvbs468rm/1**](https://data.mendeley.com/datasets/kcvbs468rm/1)

**To cite this dataset:**

**Lubetzky, Anat (2022), “Hearing Health Foundation ERG project”, Mendeley Data, V1, doi: 10.17632/kcvbs468rm.1**

6. We note that you have included the phrase “data not shown” in your manuscript. Unfortunately, this does not meet our data sharing requirements. PLOS does not permit references to inaccessible data. We require that authors provide all relevant data within the paper, Supporting Information files, or in an acceptable, public repository. Please add a citation to support this phrase or upload the data that corresponds with these findings to a stable repository (such as Figshare or Dryad) and provide and URLs, DOIs, or accession numbers that may be used to access these data. Or, if the data are not a core part of the research being presented in your study, we ask that you remove the phrase that refers to these data.

**The statement was removed and a supplementary file (S1 Table) showing the results of the analysis for 2 levels of sounds was added.**

7. Please include your full ethics statement in the ‘Methods’ section of your manuscript file. In your statement, please include the full name of the IRB or ethics committee who approved or waived your study, as well as whether or not you obtained informed written or verbal consent. If consent was waived for your study, please include this information in your statement as well.

**The statement was expanded and clarified as follows:**

**This study was approved by the Institutional Review Board of the Icahn School of Medicine at Mount Sinai (#STUDY-18-00431) and New York University Committee on Activities Involving Research Subjects (#IRB-FY2016-155). All participants first signed a written informed consent.**

8. Please include your tables as part of your main manuscript and remove the individual files. Please note that supplementary tables (should remain/ be uploaded) as separate "supporting information" files.

**Done.**

9. Please include captions for your Supporting Information files at the end of your manuscript, and update any in-text citations to match accordingly. Please see our Supporting Information guidelines for more information: [http://journals.plos.org/plosone/s/supporting-information.](https://urldefense.com/v3/__http:/journals.plos.org/plosone/s/supporting-information.__;!!BhJSzQqDqA!XycIuz7H1Nut8UzwslsRAufUGRLNua0ZSRCnaf6zTUshw5rE0s4ct8duOg8he9Ma-xg4HvHGdk2m$)

**Done.**

10. We note that Figure 1 includes an image of a participant in the study.
 
As per the PLOS ONE policy ([http://journals.plos.org/plosone/s/submission-guidelines#loc-human-subjects-research](https://urldefense.com/v3/__http:/journals.plos.org/plosone/s/submission-guidelines*loc-human-subjects-research__;Iw!!BhJSzQqDqA!XycIuz7H1Nut8UzwslsRAufUGRLNua0ZSRCnaf6zTUshw5rE0s4ct8duOg8he9Ma-xg4Hqir6ouy$)) on papers that include identifying, or potentially identifying, information, the individual(s) or parent(s)/guardian(s) must be informed of the terms of the PLOS open-access (CC-BY) license and provide specific permission for publication of these details under the terms of this license. Please download the Consent Form for Publication in a PLOS Journal ([http://journals.plos.org/plosone/s/file?id=8ce6/plos-consent-form-english.pdf](https://urldefense.com/v3/__http:/journals.plos.org/plosone/s/file?id=8ce6*plos-consent-form-english.pdf__;Lw!!BhJSzQqDqA!XycIuz7H1Nut8UzwslsRAufUGRLNua0ZSRCnaf6zTUshw5rE0s4ct8duOg8he9Ma-xg4HpoOc1Ah$)). The signed consent form should not be submitted with the manuscript, but should be securely filed in the individual's case notes. Please amend the methods section and ethics statement of the manuscript to explicitly state that the patient/participant has provided consent for publication: “The individual in this manuscript has given written informed consent (as outlined in PLOS consent form) to publish these case details”.

If you are unable to obtain consent from the subject of the photograph, you will need to remove the figure and any other textual identifying information or case descriptions for this individual.

**The 2 individuals in the figure signed the PLOS consent and the statement was added to the Methods.**

**Reviewers' comments:**

Reviewer #1: I read the manuscript entitled “Insight into postural control patterns in unilateral sensorineural hearing loss, vestibular hypo function and age-matched controls” with great interest.

I would like to acknowledge the time and effort the authors put into writing such an interesting manuscript.

**Thank you!! ☺**

Major issues:

1. Considering that the authors aimed to compare multi-sensory integration between individuals with USNHL (and no vestibular complaints), individuals with vestibular hypo function and age-matched controls, I suggest adding a few extra lines to describe and compare causes of hearing loss and vestibular dysfunction, classification of hearing loss (sensorineural or mixed/conductive hearing loss, severity of hearing loss) and vestibular dysfunction.

**The following statements were added / expanded:**

- **Included patients had either with unilateral peripheral vestibular hypofunction and no hearing loss, i.e., vestibular neuritis, or sudden idiopathic unilateral sensorineural hearing loss (USNHL).**
- **Participants in the USNHL were excluded for mixed or conductive hearing loss.**

**Details regarding the severity of HL appear in the Table 3 and description of the sample (minimum moderate unilateral HL of 41 dB 4 frequency PTA or worse).**

2. All participants in the control group reported no hearing impairment and no vestibular symptoms but it was not formally tested. This information is important and should be added to the manuscript.

**This statement was added to the results section under ‘sample’.**

3. USNHL group was not submitted to vestibular tests. This information is important and should be added to the manuscript.

**This statement was added to the results section under ‘sample’.**

Reviewer #2: This “PONE-D-22-14616” manuscript intitle “Insight into postural control patterns in unilateral sensorineural hearing loss, vestibular hypofunction and age-matched control” is a study developed to identify postural strategies in response to sensory perturbations (visual, auditory, somatosensory). The article compares multisensory integration between individuals with unilateral hearing loss (and no vestibular complaints), individuals with vestibular hypofunction and age-matched controls in a well-established protocol using virtual reality glasses where auditory stimuli were added.
I congratulate the initiative of this clinical study which, despite having an extremely restricted number of volunteers and patients in each group, is well written with a clear design that carefully used different auditory and positional sensory tests to verify the postural sway responses in unilateral hearing loss and vestibular hypofunction.

**Thank you! ☺**

Because it is a pilot study whose limitations are well-punctuated, and the conclusions only suggest possible patterns of compensatory responses that lead to future studies that can confirm this insight; this manuscript has become very extensive with figures and tables that overlap. I suggest checking the need for all figures, tables and video.

**To reduce overlap Table 4 was moved to a supplementary file (S2 Table).**

Reviewer #3: Although the manuscript is generally interesting, I have a few concerns:


1) Title:

- The title is too wordy. I would recommend describing only the main objective of the study, without including methods (e.g., age-matched controls). .

**The title was shortened as per your suggestion.**

2) Purpose of the study

- There is a small inconsistency between the purpose of the study between the abstract (..."identify postural strategies in response to sensory perturbations") and the body of the manuscript ("compare multisensory integration ... and characterize postural responses to sensory perturbations").

**We changed the purpose in the intro to make sure the wording is consistent.**


3) Material and methods:

- It is important to characterize the Hearing Loss using validated methods, such as WHO,2020 ([https://www.who.int/publications/i/item/basic-ear-and-hearing-care-resource](https://urldefense.com/v3/__https:/www.who.int/publications/i/item/basic-ear-and-hearing-care-resource__;!!BhJSzQqDqA!XycIuz7H1Nut8UzwslsRAufUGRLNua0ZSRCnaf6zTUshw5rE0s4ct8duOg8he9Ma-xg4HoXEbD81$)) or even a different one, and not only based on the asymmetry article that was referenced. Furthermore, the reference used to justify these methods is quite old

**The definition was updated to reflect most recent WHO guidelines with the relevant reference.**

- Control participants self-reported normal hearing and absence of vestibular symptoms. As they were not evaluated before the beginning of the study, this could be considered a shortcoming of your study and thereby could have compromised the validity of some of your findings. Can you further clarify?

**The following clarification was added to the limitations section:**

**While it is possible that older controls had age-related high frequency hearing loss, this study focused on significant single sided hearing loss which is highly unlikely to be undiagnosed….
…. neither group reported vestibular complaints, and both had comparable outcomes on standardized self-report assessment of balance (ABC) and vestibular dysfunction (DHI).**

- In the table it is written that the patients have a unilateral hearing loss for years and this may imply a better use of the other somatosensory cues. Patients with vestibular hypofunction have had these symptoms for a much shorter time as compared with the hearing loss, which could have deemed a more important vestibular symptomatology as compared with the hearing loss.

**Indeed, in our sample individuals with USNHL have had the condition for years whereas half of the vestibular group had symptoms for over 3 months but less than 12. This could potentially influence their ability to cope with sensory perturbations and as per your comment we now added this point to the limitations section. In vestibular research, chronic vestibular dysfunction is considered 3 months or over because spontaneous recovery is not expected beyond 3 months. Patients with single sided hearing loss however, either experience it since childhood or undergo a series of injections prior to concluding that their loss cannot be resolved.**

**Descriptively, when comparing within the vestibular group those that had symptoms less than 1 year (N=6) vs. longer than 1 year (N=6) we observed that these groups were comparable on age, DHI, ABC and all sway parameters except for DP AP on the most challenging condition (foam, dynamic vision) where the vestibular group of over 1 year had significantly less sway than the vestibular group of 1 year or under. While this observation suggests that differences in chronicity could have influenced sensory integration strategies and so should be considered in future research note that the 1 year or over group was still significantly higher than the HL group**.

- As this is a pilot study, these data (and further variables) could have been better controlled.


4) Results

- A statistical analysis could be performed between the three groups or between USNHLxVestibular groups?

**The models that we ran performed an analysis of the 3 groups using contrast coding where the control group was used as the reference. In our results, we found that the vestibular group consistently showed larger sway (COP and head) than the controls whereas the USNHL either did not differ from controls or in fact showed less change in sway with sensory perturbations. Therefore, we concluded that a direct comparison of the vestibular group to the USNHL group is not needed.**

- The results of the questionnaires are described in the tables, but I do not see them discussed in the text.

**Details were added under Results – Sample.**

5) Conclusion:

- The conclusions must be reformulated in view of the objectives.

**We shortened and reorganized the conclusion to focus directly on the purpose of this study.**
